# Advanced surface passivation for high-sensitivity studies of biomolecular condensates

**DOI:** 10.1073/pnas.2403013121

**Published:** 2024-05-23

**Authors:** Run-Wen Yao, Michael K. Rosen

**Affiliations:** ^a^Department of Biophysics, HHMI, University of Texas Southwestern Medical Center, Dallas, TX 75390

**Keywords:** phase separation, biomolecular condensate, surface passivation, self-assembly, single-molecule imaging

## Abstract

Biomolecular condensates are cellular compartments that concentrate biomolecules without an encapsulating membrane. In recent years, significant advances have been made in the understanding of condensates through biochemical reconstitution and microscopic detection of these structures. Quantitative visualization and biochemical assays of biomolecular condensates rely on surface passivation to minimize background and artifacts due to condensate adhesion. However, the challenge of undesired interactions between condensates and glass surfaces, which can alter material properties and impair observational accuracy, remains a critical hurdle. Here, we introduce an efficient, broadly applicable, and simple passivation method employing self-assembly of the surfactant Pluronic F127 (PF127). The method greatly reduces nonspecific binding across a range of condensates systems for both phase-separated droplets and biomolecules in dilute phase. Additionally, by integrating PF127 passivation with the Biotin-NeutrAvidin system, we achieve controlled multipoint attachment of condensates to surfaces. This not only preserves condensate properties but also facilitates long-time fluorescence recovery after photobleaching imaging and high-precision single-molecule analyses. Using this method, we have explored the dynamics of polySIM molecules within polySUMO/polySIM condensates at the single-molecule level. Our observations suggest a potential heterogeneity in the distribution of available polySIM-binding sites within the condensates.

## Significance

The understanding of biomolecular condensates has significantly benefited from biochemical reconstitution with microscopy detection. Here, we present a unique surface passivation method utilizing self-assembly of Pluronic F127 on hydrophobic surfaces. This approach not only effectively minimizes nonspecific binding without altering the physical properties of the condensates but also offers passivation across a variety of condensate systems. It demonstrates high resistance to different treatments and enables condensate immobilization through controlled anchor points. This allows for highly sensitive analytical techniques, including single-molecule imaging. The simplicity and high performance of this method, coupled with time and cost efficiencies, could facilitate robustness and throughput of experiments, and could broaden the accessibility of biochemical phase separation studies to a wider scientific community.

Biomolecular condensates are cellular compartments that concentrate biomolecules without an encapsulating membrane (1, 2). Many condensates form through liquid–liquid phase separation of multivalent macromolecules (1, 3). Significant advancements in our understanding of condensates have been derived from biochemical reconstitution with fluorescence microscopy detection (4–7). Undesirable interactions between condensates and glass surfaces represent a significant methodological challenge in these experiments. Such interactions can alter condensate material properties and generate appreciable background (4, 8, 9). Yet, complete inhibition of surface adhesion is also undesirable, since many experiments are impaired by condensate movement. Effective surface passivation, balancing these effects, is crucial to robust, quantitative biochemical studies of condensates.

Two commonly used passivation techniques in condensate biochemistry involve coating glass surfaces with methoxy polyethylene glycol (mPEG) and/or Bovine Serum Albumin (BSA) (4, 8). However, this method has substantial limitations. In our experience, mPEG/BSA passivation is often insufficient to prevent condensate spreading in macromolecular systems with high surface adhesion. Residual adhesion of biomolecules also produces high background that complicates high-precision studies such as single-molecule imaging (10). Additionally, mPEG passivation involves many steps and is time-consuming (10–13), diminishing robustness and reproducibility. Finally surface effects typically vary between condensates and conditions, necessitating optimization when parameters change. A straightforward, efficient and generically effective passivation method is a pressing need.

Self-assembly is a spontaneous process where molecules organize into stable formations, driven by noncovalent interactions (14). The use of surfactant self-assembly has gained widespread recognition for its ability to inhibit nonspecific biomolecule binding (15, 16). Here, we evaluated the passivation capabilities of several nonionic surfactants on hydrophobic glass surfaces, identifying Pluronic F127 (PF127) as the most effective agent in preventing nonspecific binding across both phase-separated condensates and biomolecules in dilute phases. The method exhibited excellent resilience across a broad spectrum of pH and salt conditions, while preserving the physical integrity of condensates. Combined with the Biotin-NeutrAvidin system, the method enabled condensates to be immobilized through controlled multipoint attachment to surfaces, facilitating movement-sensitive imaging, including Fluorescence Recovery After Photobleaching (FRAP) and single-molecule tracking. Using these methods, we explored the dynamics of polySIM molecules within polySUMO/polySIM condensates at the single-molecule level. Our observations suggest that the available polySIM-binding sites could have a heterogeneous distribution within the condensates.

## Results

### Screening Self-Assembled Surfactants for Glass Passivation.

**Fig. 1.**
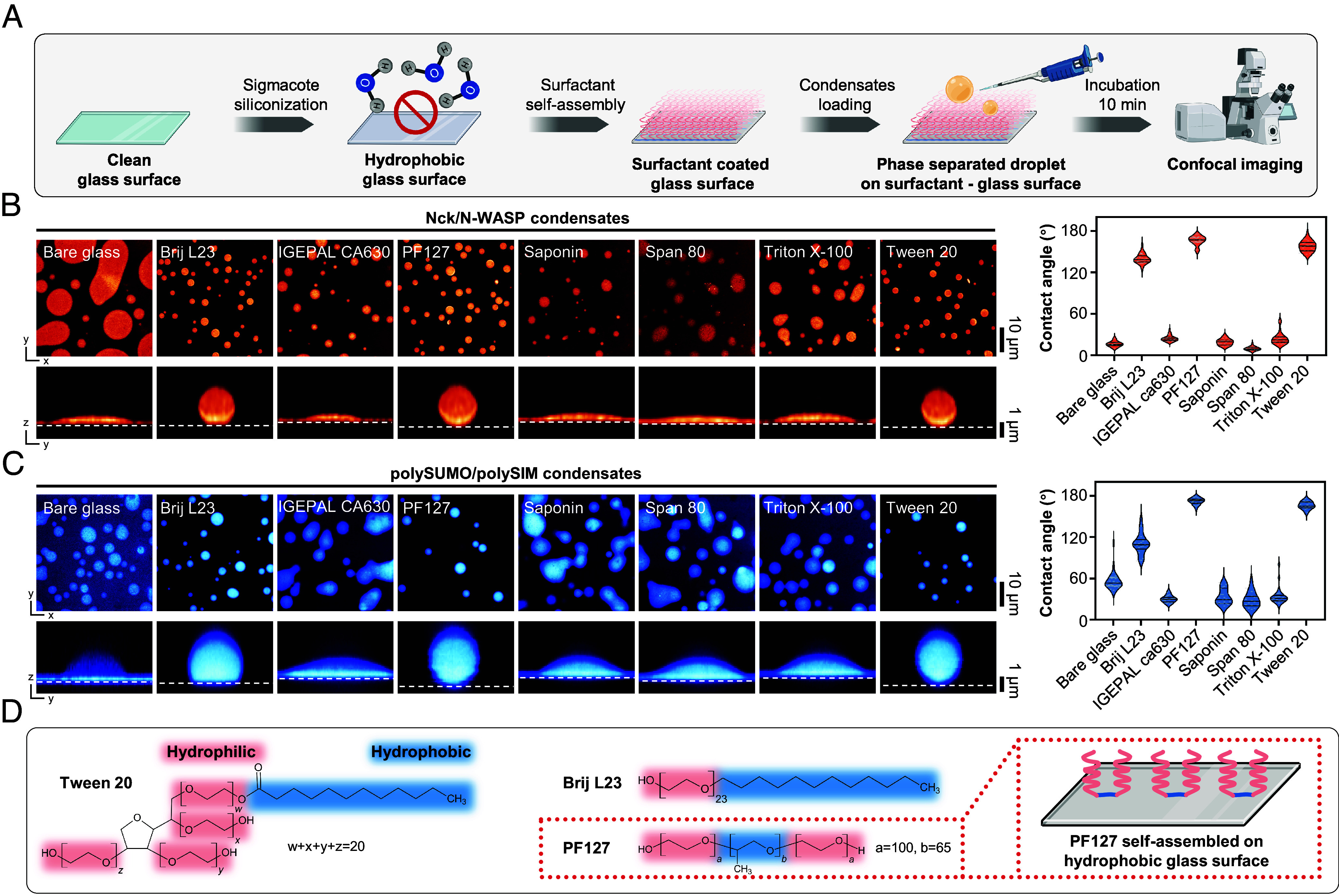
Screening self-assembled surfactants for glass passivation in phase separation studies. (*A*) Workflow illustrating the screening process for self-assembled surfactants used in glass passivation. Image created with BioRender.com. (*B*) Comparison of Nck/N-WASP condensates on different passivated surfaces. Plots show the contact angle (median and quartiles, n > 15 condensates for each technique). (*C*) Comparison of polySUMO/polySIM condensates on different passivated surfaces. Plots show the contact angle (median and quartiles, n > 15 condensates for each technique). (*D*) Chemical formula of surfactants demonstrating effective passivation abilities.

To explore the potential of self-assembled surfactants for glass surface passivation in studies of biomolecular condensates, we tested seven nonionic surfactants self-assembled on glass slides rendered hydrophobic by prior treatment with chlorinated organopolysiloxane (Sigmacote) (Fig. 1*A*). Brij L23, PF127, and Tween 20 exhibited good passivation capabilities as evidenced by the formation of phase-separated droplets with high contact angles on glass surfaces in experiments involving condensates of the actin regulatory proteins, Nck/N-WASP (6), and the engineered multivalent proteins, polySUMO/polySIM (17) (Fig. 1 *B* and *C*). These three surfactants are characterized by a hydrophobic alkyl moiety, which interacts with the hydrophobic glass surface, and one or more adjacent polyethylene oxide (PEO)-like moieties that form a dense brush layer, effectively repelling molecular binding (18, 19) (Fig. 1*D*). Notably, PF127 demonstrated the most effective passivation activity, as evidenced by the highest contact angles achieved in both condensate systems we tested (Fig. 1 *B* and *C*). Pluronic surfactants are series of commercially available triblock copolymers composed of a central polypropylene oxide (PPO) moiety flanked by PEO tails with various length. They stably attach to hydrophobic surfaces via adsorption of the PPO group and self-assembly of the PEO tails into a brush-like array, creating a hydrated layer that prevents close contact of other molecules with the glass surface (18). We further explored PF127 passivation in biochemical studies of condensates.

### PF127 Passivation Provides Effective Passivation Across Diverse Condensate Systems.

**Fig. 2.**
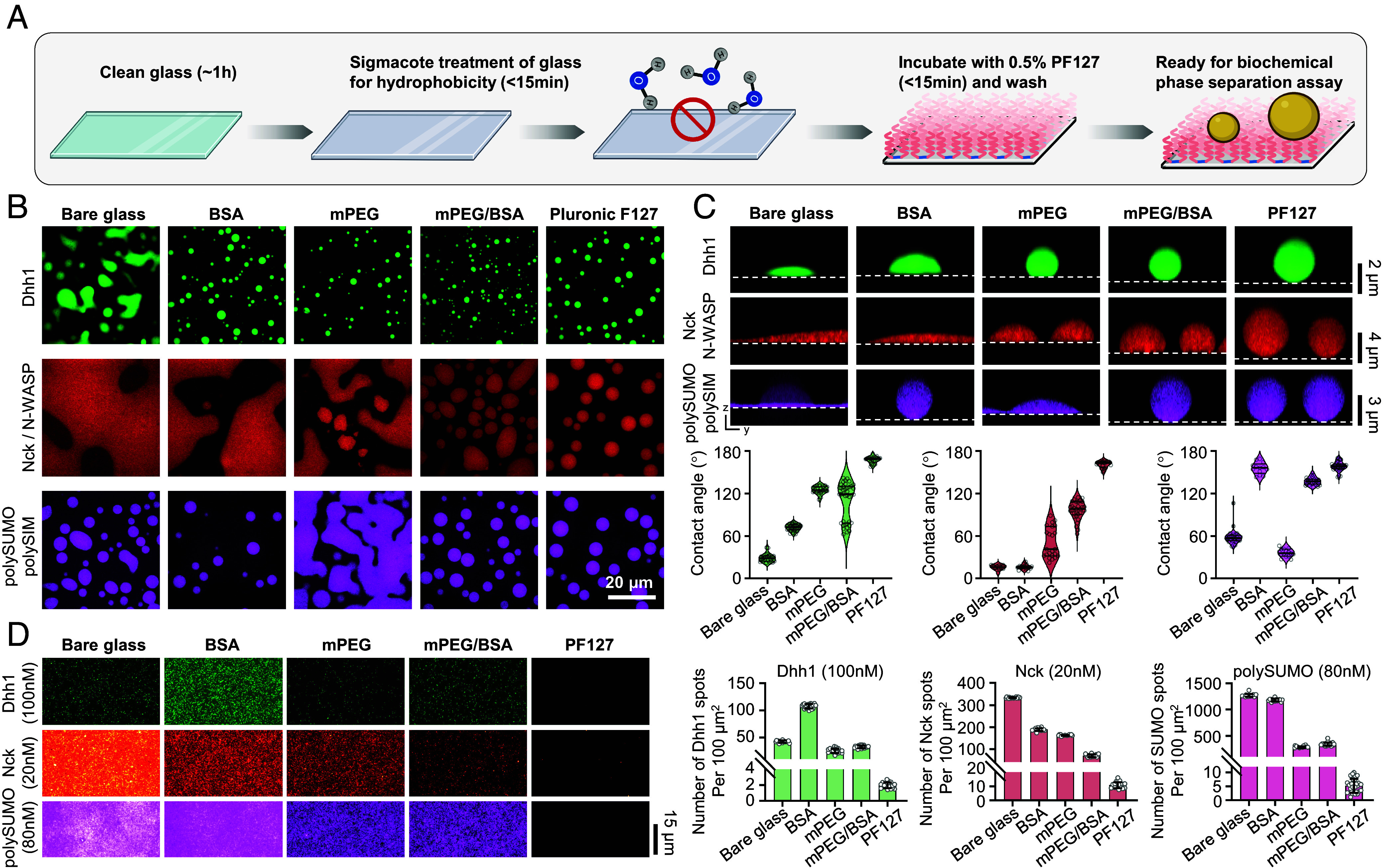
Self-assembly of PF127 as an efficient and broadly applicable surface passivation method for biochemical studies of biomolecular condensates. (*A*) Workflow illustrating PF127 passivation. Image created with BioRender.com. (*B*) Representative images showing Dhh1, Nck/N-WASP, and polySUMO/polySIM condensates on different passivated surfaces. (*C*) Comparison of PF127 passivation against traditional techniques. Plots show the contact angle (median and quartiles, n > 15 condensates per technique). (*D*) Comparison of PF127 passivation with traditional techniques at single-molecule level (mean ± SD, n > 20 images for each technique). Images for the same protein were captured under identical microscope settings and displayed with consistent brightness and contrast settings.

The PF127 passivation procedure we developed is straightforward (Fig. 2*A*, Detailed Protocol in *SI Appendix*). The entire procedure can be completed within 3 h, with active handling time <1 h (compared to >15 h for standard mPEG/BSA treatment) (*SI Appendix*, Fig. S1). We compared PF127 treatment with traditional passivation techniques, including BSA, mPEG, and combined mPEG/BSA, across three condensate systems composed of Dhh1 (7), representing IDR-mediated phase separation; Nck/N-WASP (6), generated by multivalent modular domain interactions and characterized by high wettability; and polySUMO/polySIM (17), a distinct modular domain system with low wettability. PF127 passivation yielded the lowest adhesion (highest contact angles) for all three condensates (Fig. 2 *B* and *C*). In contrast, traditional methods produced different behaviors of each condensate on differently passivated surfaces (Fig. 2 *B* and *C*).

Relatively high concentrations (10 to 1,000 nM) of free molecules often remain in the dilute phase surrounding condensates. These molecules can nonspecifically bind to slide surfaces, increasing background that impacts sensitive studies such as single-molecule imaging. To further evaluate the performance of PF127 passivation, we conducted single-molecule imaging of fluorescently labeled Dhh1, Nck, and polySUMO at concentrations mirroring the dilute phase of these systems (tens of nM) (6, 7, 17). PF127 passivation continued to demonstrate superior performance in minimizing nonspecific binding compared to traditional methods (Fig. 2*D*). Thus, for both condensates and single molecules, PF127 passivation appears to provide improved blocking of nonspecific surface adhesion.

### PF127 Passivation Is Robust Across Diverse Conditions.

**Fig. 3.**
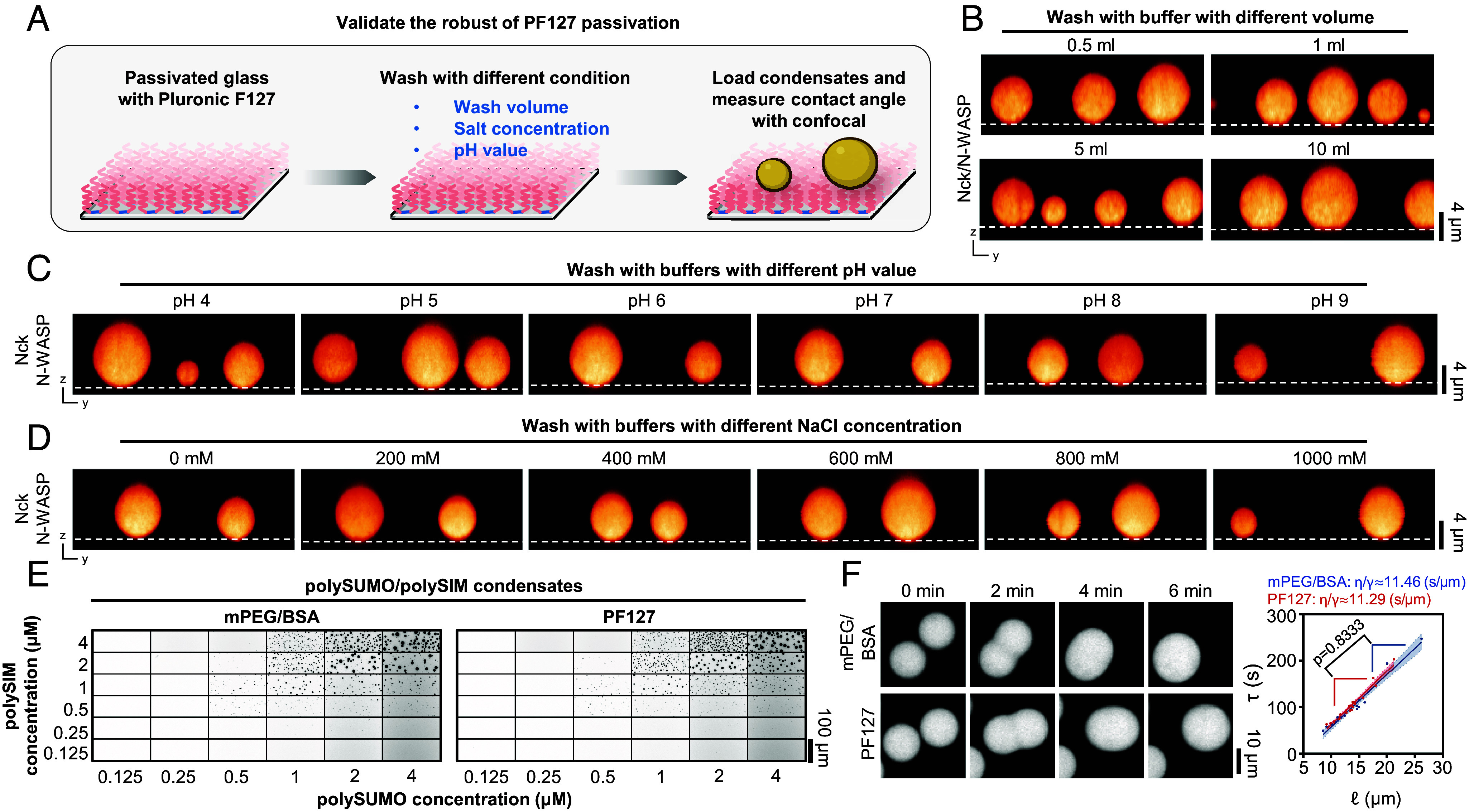
Validation of PF127 passivation robustness. (*A*) Workflow for robustness validation of PF127 passivation. Image created with BioRender.com. (*B*) Orthogonal views of Nck/N-WASP condensates on PF127-passivated surface after different buffer volume washes. Note: Imaging chamber area is 3.3 × 3.3 mm. (*C*) Orthogonal views of Nck/N-WASP condensates on PF127-passivated surface prewashed with buffers of pH values ranging from 4 to 9. The condensates were imaged under normal buffer conditions. (*D*) Orthogonal views of Nck/N-WASP condensates on PF127-passivated surface prewashed with buffer of varying NaCl concentrations (0 mM to 1,000 mM). The condensates were imaged under normal buffer conditions. (*E*) Comparison of phase separation threshold of polySUMO/polySIM condensates on glass passivated with PF127 versus mPEG/BSA. (*F*) Comparison of inverse capillary velocity of polySUMO/polySIM condensates on glass passivated with PF127 versus mPEG/BSA (mean ± 95% CI, two-tailed *t* test comparing slopes, n = 22 for PF127 and n = 17 for mPEG/BSA).

Next, we evaluated the robustness of PF127 treatment under diverse conditions: varying wash volumes, different pH values, and a spectrum of salt concentrations (Fig. 3*A*). We leveraged the strong wettability of Nck/N-WASP condensates (Fig. 2 *B* and *C*) to monitor the impact of different treatments on the integrity of passivation. Based on condensate–surface contact angles, PF127 exhibits remarkable stability. It effectively resisted 10 mL of buffer wash on a 3.3 × 3.3 mm surface area (Fig. 3*B*) and remained stable in washes of pH 4 to 9 (Fig. 3*C*) or 0 to 1 M salt prior to imaging the condensates under normal buffer conditions (Fig. 3*D*). Furthermore, imaging millimeter-scale fields of view revealed excellent homogeneity of passivation across large areas (*SI Appendix*, Fig. S2). Finally, PF127-treated surfaces displayed no autofluorescence in commonly used excitation wavelengths of 430 to 770 nm (*SI Appendix*, Fig. S3).

Even though excess free PF127 is removed during experiment procedures (Fig. 2, Detailed Protocol in *SI Appendix*) and assembled PF127 is highly stable (Fig. 3 *A*–*D*), a non-negligible concern is the potential alteration of the properties of biomolecular condensates by surfactant. To address this concern, we selected polySUMO/polySIM condensates, a system that exhibited high contact angles in both mPEG/BSA and Pluronic F127 passivation (Fig. 2 *B* and *C*), to investigate the impact of PF127 passivation on various condensate properties. PolySUMO/polySIM condensates generated on glass passivated with PF127 or mPEG/BSA showed essentially identical phase separation thresholds (Fig. 3*E*) and material properties (inverse capillary velocity) (Fig. 3*F*). Chromatin condensates exhibit variable dynamic properties depending on surface passivation (9). On PF127 passivated glass, polynucleosome arrays with 25 bp internucleosome linkers phase separated at lower concentrations than those with 30 bp linkers (*SI Appendix*, Fig. S4*A*), mirroring similar findings reported for mPEG/BSA surfaces (20). Additionally, arrays with 25 bp linkers showed recovery on ~10-min timescales in partial-droplet FRAP experiments (*SI Appendix*, Fig. S4 *B*, *Top*) and very slow material exchange with the dilute phase in whole-droplet FRAP (*SI Appendix*, Fig. S4 *B*, *Bottom*), again aligning with previous observations using mPEG/BSA surfaces (9, 20). Thus, PF127 surfaces do not aberrantly affect condensate physical properties.

### Biotin-NeutrAvidin Anchor Points Enable Biomolecular Condensates to Be Immobilized.

**Fig. 4.**
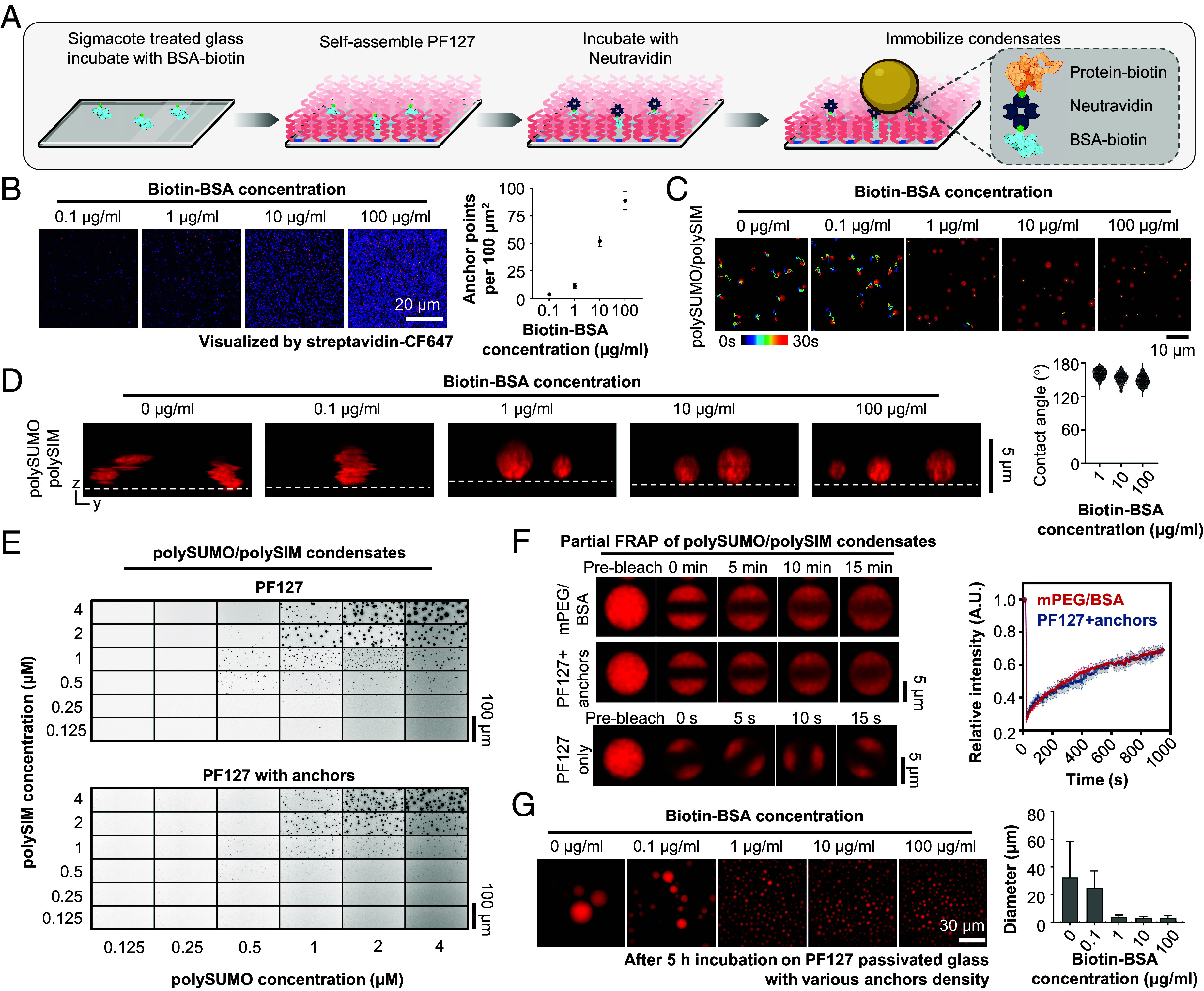
Immobilization of condensates through defined anchors. (*A*) Workflow of condensates immobilization. Image created with BioRender.com. (*B*) Representative TIRF imaging of anchor points on PF127-passivated glass incubated with varying concentrations of Biotin-BSA. Plot illustrates the density of anchor points for each concentration (mean ± SD, n > 20 images for each concentration). (*C*) Movement trajectories of polySUMO/polySIM condensates at varying biotin-BSA concentration. (*D*) Orthogonal views of polySUMO/polySIM condensates at varying biotin-BSA concentration (note that apparently irregular shapes at low biotin-BSA concentrations are due to movement during imaging of the confocal stack). Plot illustrates contact angle of polySUMO/polySIM condensates at different biotin-BSA concentrations (median and quartiles, n > 15 condensates for each condition). (*E*) Comparison of phase separation threshold of polySUMO/polySIM condensates on glass passivated with PF127 versus PF127 with anchors (1 μg/mL Biotin-BSA). (*F*) Partial FRAP of polySUMO/polySIM condensates on glass passivated by PF127 with anchor points or mPEG/BSA (mean ± SD, n = 12 condensates for each condition). For comparison, partial FRAP of polySUMO/polySIM condensates on PF127-passivated glass without anchor points is displayed at a different time resolution at the *Bottom*. (*G*) Comparison of the size of polySUMO/polySIM condensates after 5-h incubation on PF127 passivated glass surface with various anchors density. Plot illustrates the size of polySUMO/polySIM condensates for each concentration (mean ± SD, n > 100 condensates for each concentration).

A variety of investigations, including 3D imaging, microrheology, single-molecule imaging, and FRAP, benefit from condensate immobilization. However, due to the highly effective passivation ability of PF127, condensates tended to move and rotate freely across the slide surface (Movie S1). To restrain movement, we treated hydrophobic glass with 1 μg/mL Biotin-labeled BSA prior to PF127 assembly (Fig. 4*A*), to create a low density (~11 molecules/100 µm^2^) of stable, specific anchor points on the surface (Fig. 4*B*). In the presence of 15 nM NeutrAvidin, polySUMO/polySIM condensates containing 1% biotin-labeled polySUMO were completely arrested on this surface but only minimally wet it, showing contact angles of 161° ± 7° (Fig. 4 *C*–*E* and Movie S2; note that we have not yet determined the exact number of anchor points needed to effectively immobilize individual condensates). Condensate immobilization also showed no obvious effect on phase separation thresholds (Fig. 4*E*) and molecular exchange rates (Fig. 4*F*). Note that we were unable to determine the molecular exchange rates of polySUMO/polySIM condensates on PF127-passivated glass surfaces. This was due to the rapid rotation and movement of the condensates, which resulted in an unstable bleaching area and made measurements difficult (Fig. 4 *F*, *Bottom* and Movie S1). Interestingly, the immobilization of condensates impacted the size distribution of polySUMO/polySIM condensates during an extended 5-h incubation. Decreased mobility was associated with reduced condensate sizes (Fig. 4*G*). This phenomenon is likely due to reduced coalescence driven by Brownian motion, as reported previously for condensates adhered to membrane surfaces (21). Thus, PF127-treatment can produce high levels of surface passivation while simultaneously allowing condensate immobilization.

### Available PolySIM Binding Sites Are Heterogeneously Distributed within PolySUMO/PolySIM Condensates.

**Fig. 5.**
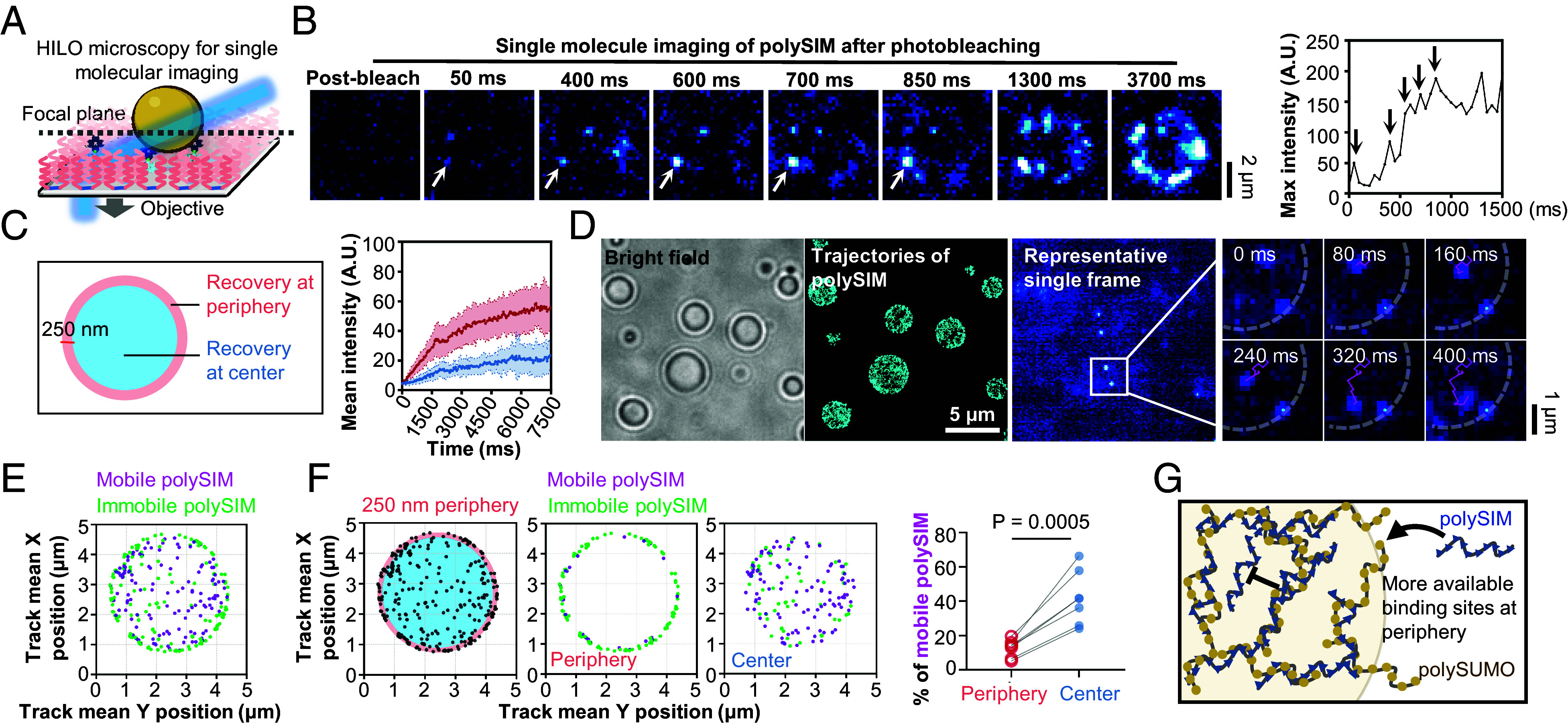
PF127 passivation facilitates high-precision single-molecule analyses. (*A*) Schematic illustration of single-molecule imaging of immobilized condensates using highly inclined and laminated optical sheet (HILO) microscopy. Image created with BioRender.com. (*B*) Single-molecule imaging of polySIM in polySUMO/polySIM condensate after photobleaching. Plot displays maximum intensity at the arrow-indicated spot. Vertical arrows within the plot mark time points at 50, 400, 600, 700, and 850 ms, correlating with the respective images on the *Left*. (*C*) Comparison of fluorescence recovery between the peripheral and central regions of condensates (mean ± SD, n = 15 condensates for each condition). (*D*) Trajectories of polySIM molecules within polySUMO/polySIM condensates (n > 2,000 tracks). *Right* image shows representative trajectories of polySIM molecules exhibiting varied mobility. The dashed line marks condensate position. The displayed area corresponds to the rectangle. (*E*) Representative distribution pattern of mobile (average velocity > 3 μm/s, magenta) and immobile (average velocity > 3 μm/s, green) polySIM molecules within condensates. Mean X and Y positions of all spots in each track are shown. (*F*) Proportions of immobile and mobile polySIM molecules at peripheral (250 nm from condensate surface) and central regions of the condensates (n = 7 condensates, paired *t* test). (*G*) Potential explanation for fewer mobile molecules in peripheral regions.

We then employed single-molecule imaging to visualize FRAP, monitoring exchange of individual fluorescent polySIM molecules between immobilized polySUMO/polySIM condensates and the dilute phase (Fig. 5*A*). Contrary to the slow overall exchange rate observed in traditional FRAP assays (Fig. 4*F*), single-molecule FRAP revealed the distinct appearance of individual fluorescence signals at the periphery of the condensates within hundreds of milliseconds after photobleaching (Fig. 5*B*). Comparisons between peripheral and central regions of polySUMO/polySIM condensates showed significantly faster recovery at the periphery (Fig. 5*C*). We first hypothesized that slow recovery at the center might be due to a low ability of new polySIM molecules to access the condensate core and slow diffusion from the periphery. However, single-molecule tracking showed that newly entered polySIM molecules readily and rapidly accessed all regions of the droplets (Fig. 5*D* and Movie S3). Analysis of the mobility patterns revealed that while polySIM molecules could access the center, many exhibited rapid diffusion there (Fig. 5 *D* and *E* and Movie S4), with an appreciably larger mobile fraction compared to the periphery (Fig. 5*F* and Movies S5 and S6). One possible explanation is that there may be a lower density of available binding sites capable of immobilizing polySIM molecules to the polymeric polySUMO/polySIM scaffold at the condensate center than at the periphery (Fig. 5*G*). Thus, availability of polySIM-binding sites, rather than accessibility, may account for the differing rates of fluorescence recovery and polySIM dynamics between the center and periphery.

## Discussion

An effective surface passivation method is crucial for preserving the material properties of biomolecular condensates, enabling accurate determination of their properties, and ensuring the success of biochemical studies. Traditional passivation methods, such as mPEG and BSA passivation, often do not sufficiently prevent nonspecific binding at concentrations used for in vitro reconstitution assays (10). This limitation makes those traditional methods suboptimal for various condensate systems, particularly those with high wettability, and fails to block adhesion of molecules to surfaces surrounding condensates, which can generate appreciable background in sensitive studies such as single-molecule imaging.

Our study introduces an efficient, robust, straightforward, and generally applicable surface passivation method via self-assembled PF127 for biochemical studies of biomolecular condensates. The method provides generic passivation for both condensates and surrounding molecules in dilute phases across diverse condensate systems, including IDR-mediated and multidomain-mediated phase separation systems with varying wettability, as well as highly sensitive chromatin condensates (Figs. 1 and 2). The method also demonstrates high resistance to extensive washing, wide ranges of pH from 4 to 9, and salt concentration from 0 to 1 M and ensures exceptional homogeneity over large areas with no autofluorescence background while preserving condensate properties (Fig. 3). When combined with a Biotin-NeutrAvidin system, the method allows condensate immobilization through specific anchor points while simultaneously producing high levels of surface passivation (Fig. 4). This facilitates multiple movement-sensitive methods that examine the bulk properties of biomolecular condensates, including 3D imaging, microrheology, FRAP, fluorescence correlation spectroscopy, and droplet fusion assays (22). This advance also paves the way for exploring molecular interactions and substructures within reconstituted condensates at the single-molecule level. In this study, we explored the dynamics of single polySIM molecules within polySUMO/polySIM condensates (Fig. 5). We achieved high temporal resolution and high localization precision, facilitated by employing our enhanced PF127 passivation technique, which reduced nonspecific background fluorescence compared to traditional mPEG/BSA passivation methods (Fig. 2), and by stably anchoring droplets to the surface without influencing their intrinsic physical properties (Fig. 4). Using single-molecule FRAP, we observed differential dynamics of newly incorporated polySIM molecules within various regions of the polySUMO/polySIM condensates: polySIM molecules in the peripheral regions exhibited slower dynamics than those in the central area (Fig. 5). One potential explanation for these preliminary observations could be the heterogeneous distribution of available polySIM-binding sites within the condensates. Nonetheless, variations in viscoelastic properties of condensates or molecular conformational heterogeneity of polySUMO across different condensate regions cannot be ruled out. Additionally, the observed higher polySIM recovery rate in peripheral regions might also suggest the influence of surface tension, which could modify the conformation, interactions, and potentially the functions of molecules within subcondensate structures (23), thereby affecting the dynamic patterns of polySIM molecules. Further research is required to decipher the mechanisms underlying the uneven molecular dynamics observed within polySUMO/polySIM condensates.

Current surface passivation methods for biomolecular condensates involve complex, time-consuming experimental procedures, sometimes requiring hazardous chemical treatments (11, 12, 24) or specialized chemical knowledge (13, 25), which diminishes their accessibility and reproducibility. In contrast, PF127 passivation is straightforward, cost-effective, and requires less than 1 h of active handling. The entire procedure can be completed within 3 h without any additional experimental expertise, offering a 99% cost reduction compared to mPEG/BSA passivation and making it feasible for any standard biochemistry laboratory. The simplicity and efficiency of the method could enhance the throughput of experiments and increase accessibility of biochemical phase separation studies to a broader scientific community.

Our method employs PF127 self-assembly to prevent nonspecific biomolecule binding and condensate wetting, necessitating only the formation of the PF127 layer on hydrophobic surfaces to create a dense, brush-like structure without covalent interactions (15, 18, 26). Thus, it can potentially passivate other experimental materials, provided they are hydrophobic, expanding its application beyond microscopic observation and reducing experimental errors caused by nonspecific binding. Furthermore, as many surfactants can self-assemble on hydrophobic surfaces (27, 28), different surfactants could create surfaces with unique properties, which could facilitate studying material properties such as surface tension, hydrophobicity, and viscoelasticity of biomolecular condensates. In future developments, mixing different surfactants might generate a rough, heterogeneous surface, which could further create a superrepellent surface for biomolecular condensates studies (29).

## Materials and Methods

### Protein Expression, Purification, and Conjugation.

#### Nck purification.

BL21(DE3) cells expressing GST-Nck were collected by centrifugation and lysed by sonication in 25 mM Tris-HCl (pH 8.0), 200 mM NaCl, 2 mM EDTA (pH 8.0), 1 mM DTT, 1 mM PMSF, 1 μg/mL antipain, 1 μg/mL benzamidine, 1 μg/mL leupeptin, and 1 μg/mL pepstatin. Proteins were affinity-purified with Glutathione Sepharose 4B (GE Healthcare). GST was cleaved from protein by TEV protease treatment for 16 h at 4 °C, followed by anion-exchange chromatography using Source 15 Q resin. Eluted protein was pooled and purified further by a Source 15 S cation exchange column. Eluted protein was concentrated using Amicon Ultra Centrifugal Filter units (Millipore) and further purified by size exclusion chromatography using a Superdex 75 prepgrade column (GE Healthcare) in 25 mM HEPES (pH 7.5), 150 mM NaCl, and 1 mM DTT.

#### N-WASP purification.

BL21(DE3) cells expressing His6-N-WASP were collected by centrifugation and lysed by cell disruption (Emulsiflex-C5, Avestin) in 20 mM imidazole (pH 7.0), 300 mM KCl, 5 mM βME, 0.01% NP-40, 1 mM PMSF, 100 μM antipain, 1 mM benzamidine, 100 μM leupeptin, and 1 μM pepstatin. Proteins were affinity-purified with NiNTA agarose (Qiagen). The eluate was further purified over a Source 15 Q column (Cytiva). The His6-tag was removed by TEV protease treatment at 4 °C for 16 h. Cleaved N-WASP was then applied to a Source 15 S column (Cytiva). Fractions containing N-WASP were concentrated using Amicon Ultra Centrifugal Filter units (Millipore) and further purified by size exclusion chromatography using a Superdex 200 prepgrade column (Cytiva) in 25 mM HEPES (pH 7.5), 150 mM KCl, 1 mM DTT, and 10% glycerol.

#### PolySUMO purification.

BL21(DE3) cells expressing His6-PolySUMO were collected by centrifugation and lysed by cell disruption (Emulsiflex-C5, Avestin) in 20 mM imidazole (pH 7.0), 300 mM KCl, 5 mM βME, 1 mM PMSF, 100 μM antipain, 1 mM benzamidine, 100 μM leupeptin, and 1 μM pepstatin. Proteins were affinity-purified with Ni-NTA Agarose Resin (Qiagen), followed by cation-exchange chromatography using Source 15S Resin (Cytiva). Purified fractions were pooled and cleaved with TEV protease and flowed through Source 15S resin to remove uncleaved proteins. Cleaved protein products were purified further by anion exchange chromatography using Source 15Q Resin (Cytiva), followed by size exclusion chromatography using a Superdex 200 gel filtration column (Cytiva) in 25 mM HEPES (pH 7.5), 150 mM KCl, 1 mM DTT, and 10% glycerol.

#### PolySIM purification.

BL21(DE3) cells expressing His6-PolySIM were collected by centrifugation and lysed by cell disruption (Emulsiflex-C5, Avestin) in 20 mM imidazole (pH 7.0), 300 mM KCl, 5 mM βME, 1 mM PMSF, 100 μM antipain, 1 mM benzamidine, 100 μM leupeptin, and 1 μM pepstatin. Proteins were affinity-purified with Ni-NTA Agarose Resin (Qiagen), followed by cation exchange chromatography using Source 15S Resin (Cytiva). Purified fractions were pooled and cleaved with TEV protease and flowed through Source 15S resin to remove uncleaved proteins. Cleaved protein products were purified further by anion exchange chromatography using Source 15Q Resin (Cytiva), followed by size exclusion chromatography using a Superdex 200 gel filtration column (Cytiva) in 25 mM HEPES (pH 7.5), 150 mM KCl, 1 mM DTT, and 10% glycerol.

#### His6-MBP-Dhh1-mEGFP purification.

BL21(DE3) cells expressing His6-MBP-mEGFP-Dhh1 were collected by centrifugation and lysed by cell disruption (Emulsiflex-C5, Avestin) in 20 mM Tris (pH 8.0), 500 mM NaCl, 10% glycerol, 10 mM imidazole, and 5 mM βME. Proteins were affinity-purified with Ni-NTA Agarose Resin (Qiagen), followed by affinity-purification with amylose resin (New England Biolabs). The amylose eluates were purified further by anion-exchange chromatography using Source 15Q Resin (Cytiva), followed by size exclusion chromatography using a Superdex 200 gel filtration column (Cytiva) in 10 mM MES (pH 7), 300 mM KOAc, 5 mM BME, and 5% glycerol.

#### Conjugation.

Recombinant proteins to be labeled with fluorophores were concentrated using Amicon Ultra Centrifugal Filter units (Millipore) to ~100 μM. Then, 5 mM βME was added to reduce cysteine residues followed by buffer exchange using a HiTrap 26/10 Desalting column (GE Healthcare) in 25 mM HEPES (pH 7.5) and 150 mM NaCl. Fractions containing protein were collected and concentrated to 100 μM. Following that, 500 μM Alexa Fluor 647 C2 Maleimide (ThermoFisher, for polySUMO), Atto 647N Maleimide (Sigma, for Nck and polySIM) or Biton Maleimide (Sigma, for polySUMO) was added, and the reaction was incubated with gentle mixing at 4 °C for 16 h. The reaction was quenched with 1 μL 14.3 M βME followed by final buffer exchange using size exclusion chromatography (GE Healthcare).

Polynucleosomal arrays were purified, assembled, and conjugated as previously described (20).

### Preparation of PF127 Passivated Surface.

#1.5H glass slides (Thorlabs) or #1.5H 384-well microscopy plates (Cellvis) were cleaned using a 1-h wash with 5% Hellmanex and thorough rinsing with H2O, followed by a 1-h etching with 1M KOH and another copious rinse with H2O (see *SI Appendix*, *Supplementary Protocol* for detailed procedures). Subsequently, the glass surfaces were treated with Sigmacote (Sigma) according to the manufacturer's instructions. The slides or plates were then rinsed with isopropanol and allowed to dry completely in a chemical hood and can be stored at this stage for up to several months.

For passivation without anchor points, slides or plates were then incubated with 0.5% PF127 (Sigma) in 20 mM Tris (pH8.0), 150 mM KCl for 15 min at room temperature. A final wash with the phase separation assay buffer was performed twice. The slides or plates were then ready for use. It is important that once treated with PF127 the slides or plates must be kept under an aqueous solution; drying irreversibly damages the self-assembled surfactant layer.

For passivation with anchor points, slides or plates were incubated with 1 to 10 μg/mL biotinylated BSA (Sigma) in 20 mM Tris (pH 8.0), 500 mM KCl for 5 min. This step was followed by a 15-min incubation with 0.5% PF127 (Sigma) in 20 mM Tris (pH 8.0), 150 mM KCl at room temperature. Afterward, the slides or plates were washed twice with 20 mM Tris (pH 8.0), 150 mM KCl and incubated with 15 nM NeutrAvidin in the same buffer for 5 min. A final wash with the phase separation assay buffer was performed twice. The slides or plates were then ready for use.

A detailed protocol is available (*SI Appendix*).

### Preparation of mPEG/BSA Passivated Surface.

mPEG passivation was performed as previously described (9). Briefly, #1.5H glass slides (Thorlabs) or #1.5H 384-well microscopy plates (Cellvis) were washed with 5% Hellmanex at 37 °C for 4 h and then rinsed copiously with H_2_O. Silica was etched with 1M NaOH for 1 h at room temperature and then rinsed copiously with H_2_O. Depolymerized Silica was covalently bonded overnight at room temperature to 20 mg/mL 5K mPEG-silane (PEGWorks) suspended in 95% Ethanol. The slides or plates were washed many times with 95% Ethanol, rinsed with copious amounts of H_2_O, and completely dried in a chemical hood over 3 to 4 h. The slides or plates were rinsed three times with phase separation assay buffer and were then ready for use.

For mPEG/BSA passivation, PEGylated glass was further incubation with freshly prepared 100 mg/mL BSA in 20 mM Tris (pH 8.0), 500 mM KCl for 30 min. The slides or plates were rinsed three times with phase separation assay buffer to remove BSA and were then ready for use.

For BSA passivation, clean and etched glass was directly incubated with freshly prepared 100 mg/mL BSA in 20 mM Tris (pH 8.0), 500 mM KCl for 30 min. The slides or plates were rinsed three times with phase separation assay buffer to remove BSA and were then ready for use.

### Biomolecular Condensates Reconstitution.

Nck/N-WASP condensates reconstitution: Nck (containing 10% Atto 647N-labeled Nck) and N-WASP proteins were mixed in 25 mM HEPES (pH 7.5), 150 mM KCl to achieve a final concentration of 10 μM, unless otherwise noted, and incubated for 1 h at room temperature before imaging.

PolySUMO/polySIM condensate reconstitution: polySUMO (containing 1% Biotin labeled polySUMO in immobilization assays) and polySIM (containing 10% Atto 647 N-labeled polySIM for conventional imaging, 1% Atto 647N-labeled polySIM for single-molecule imaging after photobleaching and 0.1% Atto 647N-labeled polySIM for single-molecule tracking) proteins were mixed in 25 mM HEPES (pH 7.5), 150 mM KCl to achieve a final concentration of 5 μM concentration unless otherwise note, and incubated for 1 h at room temperature before imaging.

Dhh1 condensate reconstitution: His6-MBP-mEGFP-Dhh1 was diluted in 10 mM MES (pH 6), 50 mM KOAc buffer, and TEV protease was added at a 1:50 molar ratio to remove the His6-MBP tag and initiate condensate formation. The reaction mixture was incubated for 6 h at room temperature before imaging.

Chromatin condensate reconstitution: Polynucleosomal arrays, featuring either 25 bp or 30 bp internucleosome linker DNA and 1% Alexa 594-labeled histone H2B, were diluted in 25 mM Tris-Acetate (pH 7.5), 150 mM KOAc, and 1 mM Mg(OAc)2 buffer to a final concentration of 1 μM. The mixture was incubated for 1 h at room temperature before imaging.

### Microscopy.

Spinning disk images were captured using a Leica DMI6000 microscope, equipped with a Yokogawa CSU-X1 spinning disk confocal scanner unit and a Hamamatsu ImagEMX2 EM-CCD camera. For high-resolution images, a Leica 100× 1.49 NA oil immersion objective was utilized (as shown in Figs. 1 *B* and *C*, 2 *B* and *C*, 3 *B*–*D*, and 4 *C*–*F* and *SI Appendix*, Fig. S4*A*). Broader field views were obtained using a Leica 20× 0.4 NA air objective (as shown in Figs. 3 *E* and *F* and 4*G*).

Confocal images were acquired on a Leica SP8 microscope equipped with a 63 × 1.4 NA oil immersion objective. For detection, the system employed either hybrid (HyD) detectors (*SI Appendix*, Figs. S2 and S3) or PMT detectors (Fig. 4*E*).

Total internal reflection fluorescence (TIRF) images were captured using a TIRF/iLAS2 TIRF/FRAP module (Biovision) mounted on a Leica DMI6000 microscope base equipped with Leica 100× 1.49 NA oil immersion objective (Fig. 4*B*) or a DeltaVision OMX SR system, equipped with an Olympus 60 × 1.49 NA oil immersion objective for TIRF and ring-TIRF capabilities (Fig. 2*D*).

Highly inclined and laminated optical sheet (HILO) microscopy for single-molecule imaging was captured using DeltaVision OMX SR system, equipped with an Olympus 60 × 1.49 NA oil immersion objective for TIRF (Fig. 5 *B*–*F*).

### Measuring Contact Angle.

To measure the contact angles, stacks of condensates were captured using a spinning disk confocal microscope with 100× objective. Spherical aberration artifacts were corrected as described previously (30). These image stacks were then processed using Fiji/ImageJ software to generate orthogonal (y-z) views of the condensates. The contact angles were subsequently measured utilizing the angle tool in Fiji/ImageJ.

### Measuring Inverse Capillary Velocity.

Time-lapse imaging of condensate fusion events was captured using a spinning disk confocal microscope equipped with 20× objective, capturing frames at intervals of 4.47 s. We selected fusion events involving two droplets of comparable size for analysis. The method for determining inverse capillary velocity was adapted from previously published protocols (31).

In brief, the aspect ratio (A.R.) of the condensates was calculated by fitting an ellipse to the shape and computing A.R. = ℓlong/ℓshort, where ℓlong and ℓshort represent the long and short axes of the ellipse, respectively. The time evolution of this aspect ratio was fit using the function A.R. = 1 + (A.R.0 − 1) × exp(−t/τ), where t is time, τ is the characteristic relaxation time, and A.R.0 is the initial aspect ratio.

For these fusing condensates, we defined the length scale as the geometric mean ℓ = [(ℓlong(t = 0) − ℓshort(t = 0)) × ℓshort(t = 0)]1/2. The relationship between τ and ℓ was characterized by fitting the data to a linear equation τ = (η/γ) × ℓ, from which we determined the inverse capillary velocity, η/γ.

### Validation of PF127 Passivation Robustness.

To validate the robustness of PF127 passivation, #1.5H 384-well microscopy plates (Cellvis) were first passivated by PF127 as described above. The wells then underwent various treatments to test the stability of the passivation:

1. Washing with varying volumes (0.5, 1, 5, and 10 mL) of 20 mM Tris (pH 8.0), 150 mM KCl buffer.

2. Incubation with 100 μL of different buffers for 10 min at room temperature: 20 mM MES (pH 4.0), 20 mM MES (pH 5.0), 20 mM MES (pH 6.0), 20 mM HEPES (pH 7.0), 20 mM Tris (pH 8.0), and 20 mM Tris (pH 9.0).

3. Incubation with 100 μL of 20 mM HEPES (pH 7.5) containing varying concentrations of NaCl (0, 200, 400, 600, 800, and 1,000 mM) for 10 min at room temperature.

Following these treatments, the wells were washed twice with the buffer used for condensate reconstitution. Condensates were then loaded into the wells for imaging.

### Characterization of Autofluorescent of PF127 Passivation.

To evaluate the autofluorescent properties of PF127 passivation, #1.5H 384-well microscopy plates (Cellvis) were first passivated by PF127 as described above. After passivation, each well was filled with 50 μL of 0.5% PF127 solution for imaging purposes. For a bare glass control, 50 μL of water was added directly into untreated wells. For comparing autofluorescence against real fluorescence level, Pacific Blue, Alexa 488, Alexa 555, and Alexa 647 dyes at concentrations representing the dilute (10 nM) and dense (10 μM) phases were added into untreated wells. Imaging was conducted using a Leica SP8 microscope using the λ scanning function. Excitation was performed using 405 nm, 488 nm, 552 nm, and 638 nm lasers at 5% laser power, while emission light ranging from 430 to 770 nm was collected in 20 nm bandwidth increments by HyD detector at 100% gain.

### FRAP.

FRAP assays were performed using a Leica SP8 microscope equipped with a 100 × 1.4 NA oil immersion objective (Fig. 4*F*) or a DeltaVision OMX SR system equipped with a 60 × 1.42 NA oil immersion objective and ring-TIRF capabilities (*SI Appendix*, Fig. S4*B*).

In these assays, a designated region of interest (ROI) was subjected to photobleaching, and the subsequent recovery of fluorescence intensity within the ROI was monitored and recorded for each experiment. The intensity recovery curves were then normalized and corrected for photobleaching effects, following methodologies described previously (32). Briefly, the fluorescence signal measured in a ROI normalized to the change in total fluorescence was determined as I = (It/I0) × (T0/Tt), where I0 is the average intensity of the unbleached ROI at time point t. I0 is the average prebleach intensity of the ROI. T0 and Tt are the average fluorescence intensities of a neighboring condensate in the same field of view at prebleach or at timepoint t, respectively. The recovery curves were fit to the following expression using GraphPad as Y(t) = A (1-e(τ×t)), where A is the end-value of the recovered intensity, τ is the fitted parameter, and t is the time after the bleaching.

### Single-Molecular Tracking and Analysis.

Single-molecule imaging was performed using a DeltaVision OMX SR system, equipped with a 60 × 1.49 NA TIRF oil immersion objective and ring-TIRF system. Imaging was conducted in epi/TIRF mode using CMOS cameras set to 2 × 2 binning and a beam condenser to enhance contrast.

For single-molecule imaging after photobleaching (refer to Fig. 5 *B* and *C*), the photokinetic mode was employed to bleach the condensates, and signal recovery was captured at a time resolution of 50 ms. A total of approximately 150 images (~7.5 s) were recorded for each event. The images were processed using Fiji/ImageJ for background subtraction. The mean intensity in different regions of the condensates (periphery and center) was measured and plotted.

For single-molecule tracking of newly entered polySIM (refer to Fig. 5 *D*–*F*), the burst mode was used at 50 Hz imaging rate. Movies were first processed using Fiji/ImageJ for background subtraction. The first 100 frames of each movie were discarded to ensure that only newly entered polySIM molecules were imaged. The movies were then analyzed using TrackMate in Fiji/ImageJ. Specifically, spots were detected using the Hessian detector with a 550-nm diameter and subpixel localization. Spots were then filtered based on contrast, quality, signal-to-noise ratio, and maximum intensity. Track generation was accomplished using the LAP Tracker with a maximum frame-to-frame linking distance of 500 nm and a maximum gap of 2 frames, also at a maximum distance of 500 nm. Tracks consisting of at least 6 events were retained, and trajectories were plotted. The average velocity of tracks was used to categorize the events: tracks with average velocity <3 μm/s were classified as immobile, while those >3 μm/s were classified as mobile. The mean of the X and Y position of all the spots in each track was used to indicate the position of polySIM molecules. The number of polySIM in each mobility category was counted in both the peripheral and central regions of the condensates.

## Supplementary Material

Appendix 01 (PDF)

Movie S1. Rotation and free movement of polySUMO/polySIM condensates across the PF127 passivated glass surface. Rectangular photo-bleached areas were used to observer rotation.

Movie S2. Movement of polySUMO/polySIM condensates on PF127 passivated glass surface with different densities of anchor points, related to Fig. 4C.

Movie S3. Trajectories of polySIM molecules within polySUMO/polySIM condensates, related to Fig. 5D. Images were captured at a 50 Hz rate. The playback speed is ten times the actual speed.

Movie S4. Representative trajectories of mobile and immobile polySIM molecules within polySUMO/polySIM condensates, related to Fig. 5D.

Movie S5. Trajectories of immobile polySIM molecules within polySUMO/polySIM condensates, related to Fig. 5E and 5F. Images were captured at a 50 Hz rate. The playback speed is ten times the actual speed.

Movie S6. Trajectories of mobile polySIM molecules within polySUMO/polySIM condensates, related to Fig. 5E and 5F. Images were captured at a 50 Hz rate. The playback speed is ten times the actual speed.

## Data Availability

Original data have been deposited in Mendeley Data (DOI: https://doi.org/10.17632/t4yjmnngs9.1) (33).
